# Successful Pavlik treatment in late-diagnosed developmental dysplasia of the hip

**DOI:** 10.1007/s00264-012-1587-5

**Published:** 2012-06-12

**Authors:** Michiel A. J. van de Sande, Frank Melisie

**Affiliations:** Department of Orthopedics, Leiden University Medical Center, J11-R70, PO Box 9600, 2300 RC Leiden, The Netherlands

## Abstract

**Purpose:**

The objective of this study was to evaluate success and complication rates of the Pavlik harness in late-diagnosed hip dislocation (developmental dysplasia of the hip, DDH). We also set out to assess the additional value of an abduction brace for three to six months, when successful reduction using the Pavlik harness was achieved.

**Methods:**

We included 31 patients (31 hips, 28 female, right/left = 4/27) with late-diagnosed hip dislocation treated with a Pavlik harness between 1995 and 2008. The average age at the start treatment was 27 weeks (21-57). None were lost to follow-up; the mean follow-up was 4.2 years (two-10). Of these patients, 61 % were classified as Tönnis type 2, 32 % as type 3 and 7 % as type 4.

**Results:**

Of 31 hips, 20 (65 %) were successfully reduced after the use of the Pavlik harness. The average duration of Pavlik treatment was seven weeks (three-12). The mean time to stable reduction was six weeks (three-11), after which the patients were weaned off the Pavlik harness. The mean age at final treatment was 19 months (12-28). Five patients (15.0 %) developed radiological signs of osteonecrosis (Kalamchi and MacEwen classification; two group I, one group II and two group IV). When we compared the different Tönnis types a significant difference in successful reduction was found. Seventeen (81 %) of Tönnis type 2 dislocated hips were successfully reduced, while only two (25 %) of Tönnis type 3-4 hips were (odds ratio 25, *p* = 0.001). Clinical examination (e.g. limited abduction, positive Ortolani, Barlow or Galeazzi) at the time of diagnosis was not significantly related to the success rate of the Pavlik treatment. The mean acetabular index (AI) significantly improved from 36.5 to 30.5° after initial Pavlik treatment to 22.3° at final follow-up (4.1 years).

**Conclusions:**

Prolonged use of the Pavlik harness in late-diagnosed hip dislocation (DDH) Tönnis type <3 is a safe and successful treatment option in the older infant. Although the AI was significantly reduced after the abduction brace, its additional use remains debatable, as no control group was evaluated.

## Introduction

Although late-diagnosed hip dislocations are now rarely seen because of national screening programmes, they still present a significant challenge in their management and literature supporting any treatment protocol remains scarce [2, 10]. In The Netherlands newborns at risk, or after a positive clinical examination, are to be screened with ultrasound at six–12 weeks as timely intervention can then be managed with a Pavlik harness, possibly followed by a Camp abduction brace if the result after Pavlik treatment is not satisfactory [7]. Still a fair number of children (∼30 %) present at our clinic with a late-diagnosed hip dislocation (>five months). Clinical findings in late presenting hip dislocation are often only limited abduction and leg length discrepancy, as muscle tone and decreased capsular laxity prevent a reliable clinical differentiation to be made between a dislocated and subluxated hip [1, 2]. Preferred imaging studies in these older children are either ultrasound, which is best used up until 4.5 months of age while the femoral head is largely cartilaginous, or plain anteroposterior (AP) pelvic radiographs [12]. Commonly advised treatment for a late-diagnosed hip dislocation is a closed reduction followed by spica casting, possibly resulting in short hospitalisation, additional X-rays (CT scans) or ultrasounds [4]. Only a few studies have reported on the success and complication rates for the use of the Pavlik harness in older infants [2, 10].

The objective of this study was to evaluate the results and complications of Pavlik harness treatment in older infants with a late-diagnosed hip dislocation. The additional value of the Camp abduction brace after successful reduction using the Pavlik harness in this older patient population was also evaluated.

## Patients and methods

Between 1995 and 2008, 109 patients (118 hips) were treated in our hospital with a Pavlik brace for developmental dysplasia of the hip (DDH) with a dislocated or subluxed hip. Patients were excluded when follow-up was less than two years or when the child was younger than five months of age at the start of the treatment. Also teratological DDH cases and patients primarily treated outside our hospital were excluded. This left us with 31 infants (31 hips, 28 female, right/left = 4/27) that were diagnosed after the age of five months and treated at our department using a Pavlik harness. The average age of the children at the start of the Pavlik harness treatment was 27 weeks (21–57 weeks). None were lost to follow-up, with a mean follow-up of 4.2 years (two to ten years).

Interestingly 14 children did not present with any risk factors for DDH (45 %); seven were born in a breach position (22 %); of these five were frank breach and six were delivered by caesarean section. Ten children had close family members with DDH (31 %).

Clinical examination at two to four weeks after birth was not available for the majority of these children. Clinical data were available in only nine children for the first clinical screening at two to three weeks. Of these children only three presented with limited abduction; none were described as dislocated or unstable. All presented with risk factors and were therefore screened and diagnosed using radiography at five months. Clinical examination was performed at first presentation in our hospital between 21 and 57 weeks. In five children the hip examination was described as normal, eight showed only limited abduction, ten limited abduction and a positive Galeazzi sign and another four were described as limited in abduction with a positive Barlow sign. Although described as unreliable after the age of three months, positive Ortolani and Barlow manoeuvres were observed in five hips.

### Treatment protocol

All patients were diagnosed with congenital hip dislocation using plain AP radiographs of the pelvis, as radiographs in infants older than five months were believed to be more reliable at this age when compared to ultrasound evaluation.

In all children initial treatment consisted of an attempt at closed reduction using a Pavlik harness. After one and two weeks the infants were examined to determine whether the harness had to be adjusted in order to achieve proper flexion (∼ 100°) and abduction (∼ 70°). Every three to four weeks a repeat radiograph with and without the Pavlik harness was made to assess if the hips were reduced or unstable reduction was managed. It was then decided whether the Pavlik treatment was to be continued or a closed (or open) reduction under general anaesthesia was necessary in case of persistent dislocation. If reduction was stable, but residual acetabular dysplasia was noted on the final X-ray, the hip abduction treatment was continued using a Camp abduction brace and Pavlik treatment was discontinued. The switch from Pavlik to Camp within this time frame was supported by the thought that after stable reduction was managed using the Pavlik harness the residual dysplasia could now safely be treated with a more patient (parent) friendly abduction device. The prolonged use of the Pavlik harness was based on reports of successful reduction after the prolonged use of the Pavlik harness by van der Sluijs et al. and Pollet et al. [10, 13].

**Fig. 1:**
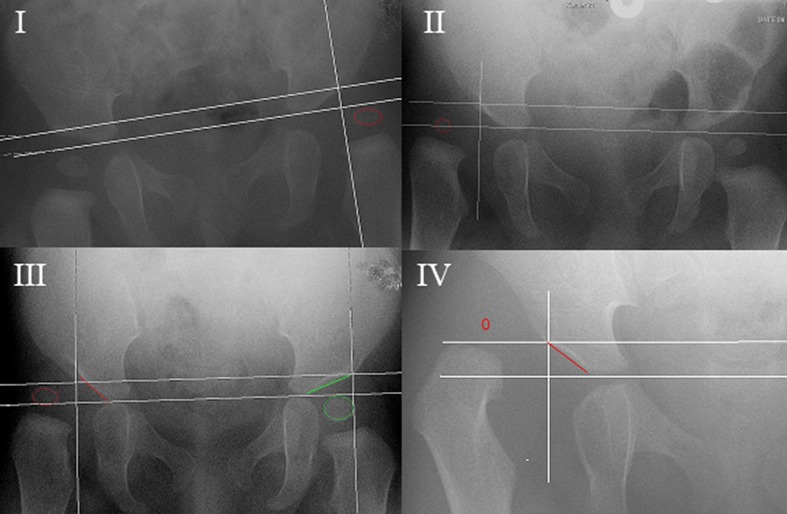
Modified Tönnis classification [6]. *I* type 1: capital femoral epiphysis lateral to Perkin’s line but below the level of the inferior acetabular rim, *II* type 2: capital femoral epiphysis lateral to Perkin’s line but below the level of the superior acetabular rim, *III* type 3: capital femoral epiphysis at the level of the superior acetabular rim and *IV* type 4: capital femoral epiphysis above the level of the superior acetabular rim

**Fig. 2:**
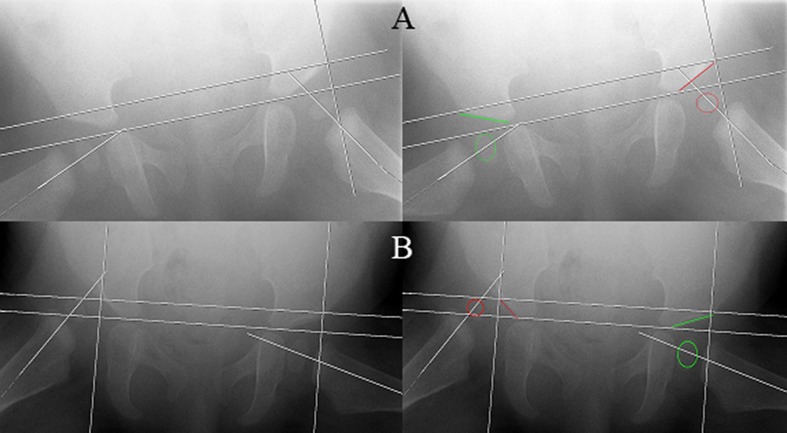
Frog-leg or Lauenstein AP radiograph. **a** The femoral head is positioned centrally within the true acetabulum with the hips flexed and abducted; this hip is regarded as subluxed as the femoral neck is not directed towards the triangular cartilage. **b** With hips flexed and abducted the femoral head remains positioned lateral to Perkin’s line and superior to the acetabular rim; this hip is regarded as dislocated

X-rays were assessed at initial diagnosis, after the end of treatment with the Pavlik harness, at the end of hip abduction treatment and at final follow-up. To classify the hips we used the system from the Commission for the Study of Hip Dysplasia (Tönnis classification). This system is based on the position of the capital femoral epiphysis to Perkin’s line which goes vertical through the superolateral margin of the acetabulum (Fig. 1) [14]. We made use of the modified Tönnis classification [14]: type I capital femoral epiphysis lateral to Perkin’s line but below the level of the inferior acetabular rim, type II capital femoral epiphysis lateral to Perkin’s line but below the level of the superior acetabular rim, type III capital femoral epiphysis at the level of the superior acetabular rim and type IV capital femoral epiphysis above the level of the superior acetabular rim. A successfully reduced hip was described as a Tonnis type <1 or no residual subluxation on the AP radiograph (Fig. 1). Additional Lauenstein or frog-leg radiographs had to present normal positioning of the hips with the femoral neck directed towards the triangular cartilage (Fig. 2).

**Fig. 3:**
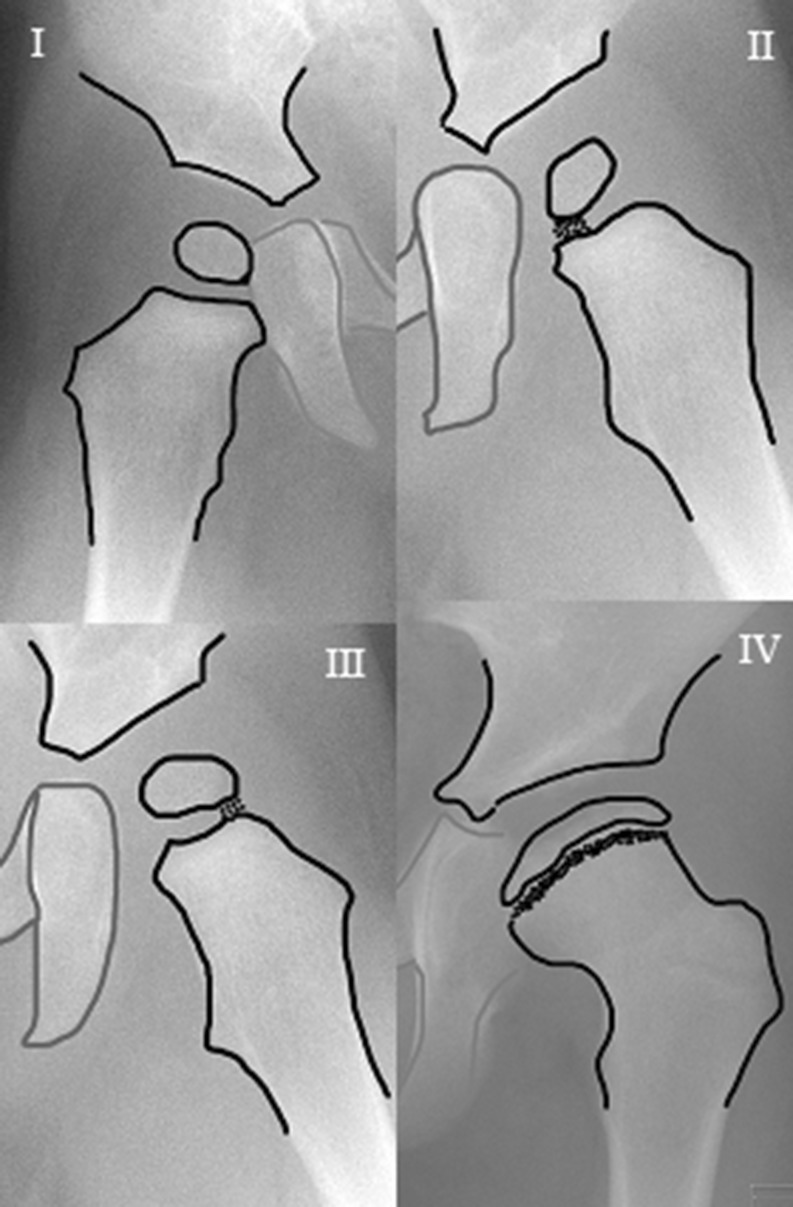
The Kalamchi and MacEwen classification: group I delayed appearance or mottling of ossific nucleus; group II lateral physeal damage; group III central physeal damage; group IV damage to the femoral head and the physis

**Fig. 4:**
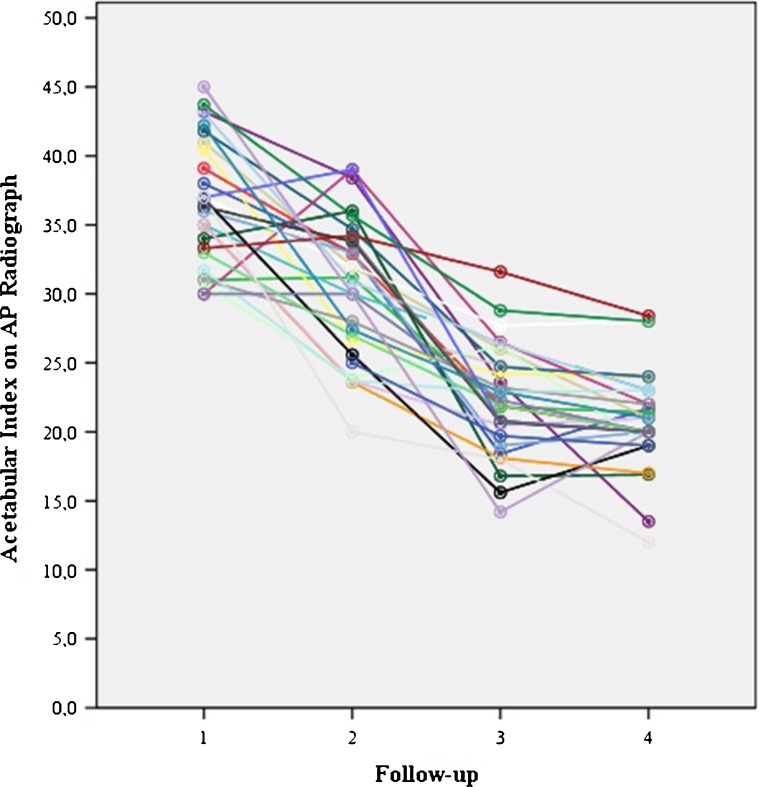
Follow-up: 1 start Pavlik (mean 6 months), 2 start Camp/Spica (mean 8.7 months), 3 end Camp/Spica (mean 19 months), 4 final follow-up (4.1 years)

**Fig. 5:**
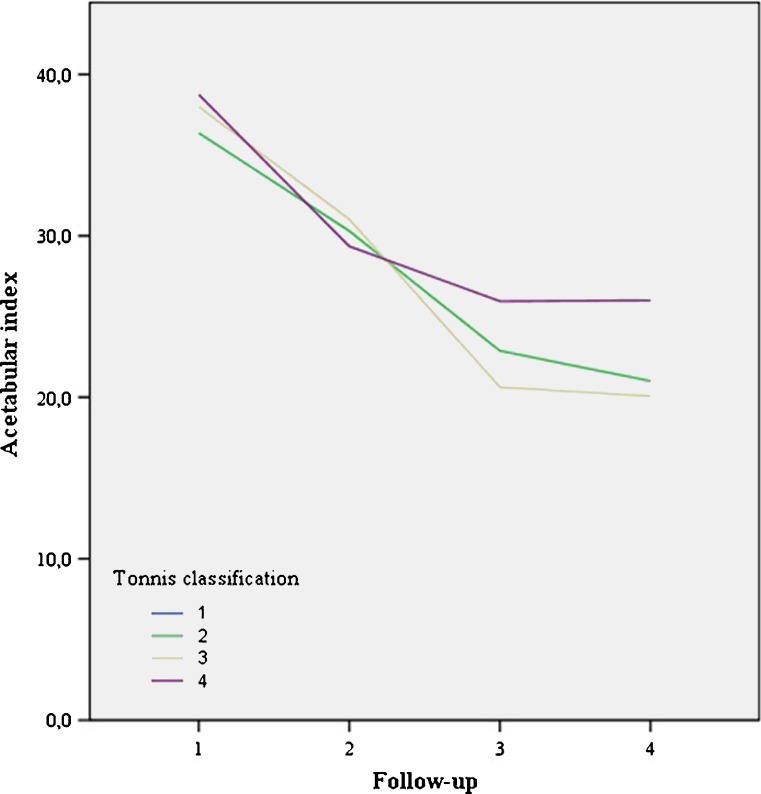
No significant difference for acetabular index: before treatment, after reduction, after Camp treatment and at final follow-up for different Tönnis types*. Follow-up: 1 start Pavlik (mean 6 months), 2 start Camp/Spica (mean 8.7 m), 3 end Camp/spica (mean 19 m), 4 final FU 4.1y). *19 hips were diagnosed as type 2, 10 as type3 and only 2 hips were diagnosed as type 4

To evaluate residual dysplasia the acetabular index (AI) was measured on all AP radiograph. AI measurements performed by the radiologist were repeated by a blinded experienced orthopaedic surgeon to evaluate the inter-observer variance. The Kalamchi and MacEwen classification was used to diagnose osteonecrosis (ON) [8]. This classification differentiates between four types of ON. Group I represents changes that solely affect the ossific nucleus; either delayed appearance or mottling is present. Group II represents lateral physeal damage; lateral ossification or irregularity and bridging or a lateral metaphyseal defect can be seen. Group III presents with central physeal damage (comparable to group II). In group IV the most severe cases present with damage to the femoral head and the physis; this type of ON often presents with varus, flattening and coxa magna (Fig. 3).

**Fig. 6:**
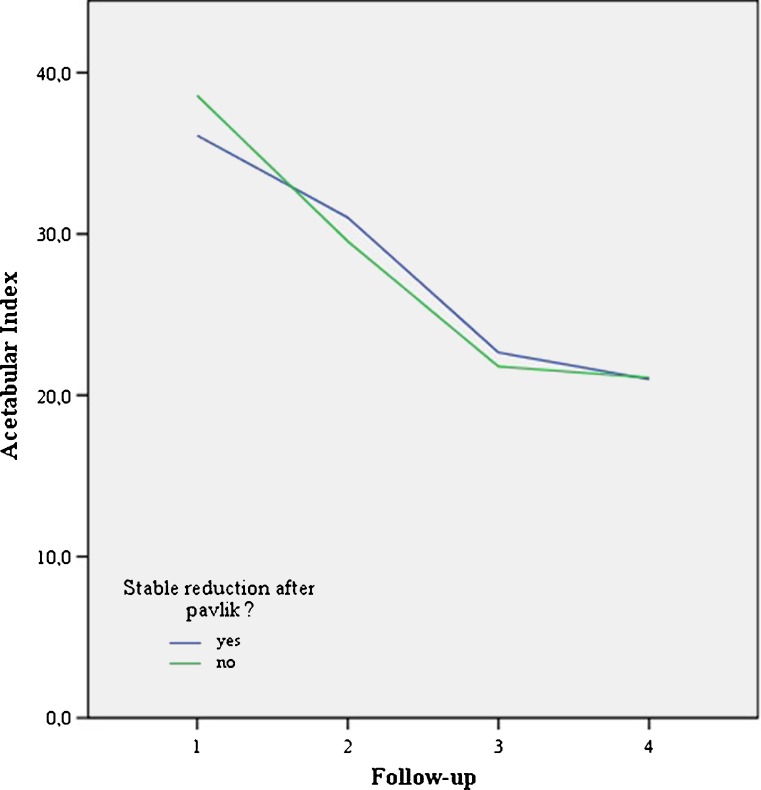
No significant difference for acetabular index: before treatment, after reduction, after Camp treatment and at final follow-up for successful and unsuccessful reduction after Pavlik treatment. Follow-up: 1 start Pavlik (mean 6 months), 2 start Camp/Spica (mean 8.7 m), 3 end Camp/spica (mean 19 m), 4 final FU 4.1y)

### Statistical analysis

In order assess the success rate of the prolonged Pavlik treatment in older infants with congenital hip dislocation we identified the need for a closed or open reduction as the endpoint identifying treatment failure. Odds ratios (OR) for successful reduction were calculated comparing Tönnis type 2 and 3-4 hips, gender, side and risk factors for DDH. A Mann-Whitney U test was used to compare Tönnis type 2 and 3-4 hips for the AI after Pavlik treatment. Furthermore, a kappa value was calculated in order to correlate the investigators measurements of the AI to the measurements done by the radiologist. Finally, the additional effect of the abduction brace was evaluated as the difference in AI directly after Pavlik treatment and at end of (abduction brace) treatment using a linear mixed model. A two-sided *p* value of <0.05 was considered significant.

## Results

On initial AP radiographs 19 hip dislocations (61 %) were classified as Tönnis type 2, ten as type 3 (32 %) and two as type 4 (7 %). In all children a Lauenstein or frog-leg radiograph of both hips was also performed at the time of diagnosis: 23 hips were not positioned centrally within the true acetabulum with the hips flexed and abducted and were described as dislocated, and 8 were described as subluxed, as the femoral head was positioned centrally but not contained within the true acetabulum.

Of 31 hips, 20 (65 %) were successfully reduced after the use of the Pavlik harness. The average duration of initial treatment with a Pavlik harness was 6.7 weeks (range three to 11 weeks). The mean time to stable reduction was six weeks (three to 11), after which the Pavlik harness was followed up by a Camp abduction brace in all but one case. In this case the child was successfully weaned off the Pavlik harness after ten weeks. Children not responding to the Pavlik treatment with a stable reduction and who were eventually treated with closed reduction and spica cast under general anaesthesia were treated with the Pavlik harness for a mean of 7.45 weeks (range four to 12 weeks). The mean age after final treatment (including spica cast immobilisation) was 19 months (range 12–28 months).

Eleven hips that failed the Pavlik treatment were all reduced by closed reduction under general anaesthesia and all patients remained successfully reduced in the spica cast that was continued for three months. All but one patient were weaned off the abduction spica using the Camp abduction brace for an additional siz to 12 weeks.

Five patients (15.0 %) developed radiological signs of ON using the Kalamchi and MacEwen classification for ON (two group I, one group II and two group IV) [8]. Group IV ON was not seen after the successful use of the Pavlik harness, only in children needing additional closed reduction and spica cast immobilisation. No other complications were noted, e.g. no femoral nerve palsy was diagnosed.

Clinical examination (e.g. limited abduction, positive Ortolani, Barlow or Galeazzi) at first presentation or at the time of diagnosis was not significantly related to the success rate of the Pavlik treatment.

A significant difference in percentages of successful reduction between the different Tönnis types was found. In type 2, 17 hips (81 %) were successfully reduced. This was significantly higher when compared to the two hips (25 %) that were successfully reduced in Tönnis type 3-4 hips (OR 25, *p* = 0.001). Additionally, the differentiation between a subluxed and dislocated hip using the Lauenstein or frog-leg radiograph at the time of diagnoses was a strong predictor of Pavlik success as all patients with a subluxed hip could be reduced using the Pavlik harness [OR 1.96 (1.3–2.8)].

The mean AI significantly improved from 36.5 to 30.5° after initial Pavlik treatment to 22.3° at final follow-up (mean 4.1 years) (Fig. 4). There was no significant difference in AI at any moment in follow-up between Tönnis type 2 and 3-4 or successful vs unsuccessful reduction groups (Figs. 5 and 6). No significant differences in AI or successful reduction were found using a mixed linear model between male and female patients or right and left hips.

AI measurements by the investigator set against measurements by the radiologist gave a correlation kappa of 0.920 (*p* < 0.001) for the pretreatment radiographs and of 0.916 (*p* < 0.001) at the end of the treatment protocol using the Camp abduction brace.

## Discussion

Over 50 years ago Arnold Pavlik published his functional method for treatment of hip dislocation in young infants [9]. Although this method successfully decreased the high rates of ON reported after closed reduction under general anaesthetics and spica cast immobilisation, its initial use was limited to smaller or younger infants as early publications advised that this treatment be discontinued in cases of late presentation, no adequate sense of reduction after two weeks, hips in need of excessive flexion or re-dislocation with slightest adduction [6, 11]. Recent reports however have presented successful reduction (>60 % in Graf type III) in children over six months [10]. Additionally, prolonged use, even if a sense of reduction is not yet achieved after two to four weeks, has also been indicated as safe and successful [6, 10]. Today, the Pavlik harness is therefore considered the standard method of treatment for DDH in young infants. This study was set up to evaluate the success and complication rates in the treatment of late-diagnosed hip dislocation in infants older than five months.

In concurrence with the very few other studies published on the subject, use of the Pavlik harness in the late-diagnosed mild hip dislocation (< Graf type IV, < Tönnis type 3) should be considered a successful treatment option in the older infant [1]. Prolonged use of the Pavlik device however is only advised in case of increased possible hip abduction or radiographic support (ultrasound in younger infants) of improved acetabular coverage [13].

There are several possible study limitations to this retrospective study on the late treatment of congenital hip dislocation. Primarily the use of pelvic radiographs may limit the extrapolation of our results to general practice, as ultrasound evaluation of this condition is considered the gold standard in the screening and diagnostics of congenital hip dislocation. However, the use of ultrasound in older infants is still considered of great value as ossification of the femoral head may limit the use of ultrasound in these children. In addition, ultrasound has not yet found its way into all paediatric clinics and significant experience in the appreciation of ultrasound images is of great influence on its reliability [12]. The high kappa value for inter-observer variance in the evaluation of DDH using radiographs as presented in this study again underlines the value of radiographic evaluation of DDH in older infants. We did not calculate a intra-observer variance, as the inter-observer variance was appreciated as good with a kappa of 0.920 (*p* < 0.001) for the initial radiograph and an identical kappa could be anticipated for the intra-observer variance.

The retrospective character and limited number of patients may induce a type 2 error in our statistical analysis. Still this study presents results on the largest number of patients treated with a Pavlik harness for congenital hip dislocation after the age of five months. This retrospective character also limited our uniformity in clinical examination at screening age (between two and six weeks) and at initial presentation and diagnosis (between 21 and 57 weeks). Clinical results (e.g. limited abduction, positive Ortolani, Barlow or Galeazzi) therefore could not be used to predict successful treatment.

None of the included patients were diagnosed with bilateral DDH. This may have introduced inclusion bias as it appears that patients with bilateral DDH were either excluded as they did not meet the age limit or were not initially treated with a Pavlik device but by primary reduction and spica cast under general anaesthesia.

Finally, the lack of a control group that was not additionally treated with an abduction device (Camp) prevented us from differentiating between the natural history of reduced hip using a Pavlik harness and the influence of this additional abduction treatment on the normalisation of the AI at final follow-up [3, 7].

The relatively few complications were only found after closed/open reduction and spica treatment [10]. In our study 15.0 % of the patients developed radiological signs of ON. According to general opinion, only group II or more is considered clinically relevant due to its irreversible changes to the shape of femoral head [8]. If we consider only these cases only three cases of ON remained (9.6 %). This is largely comparable to the percentage found by Pollet et al. (12.5 %) in their study on the late treatment of congenital hip dislocation. Bolland et al. found a rate of 11.2 % ON in a population which received closed or open reductions after failed initial treatment or late-diagnosed dislocations, also comparable to our results [1]. The percentage of re-dislocations and need for concomitant osteotomies however was much lower in our study (none needed in our population) when compared to 12.5 % in the study by Pollet et al. This difference may be explained due to the difference in age at the start of treatment (1.5 months) in favour of our study population [10, 11]. Both the risk for ON and the risk for re-dislocation and associated need for pelvic osteotomies are probably negatively influenced by the age of the patient at the start of the treatment and the severity of the hip dislocation [6, 11]. This again underlines the necessity for early detection and start of treatment. Additionally, this study underlines the possibly positive effect of the initiation of treatment using a Pavlik harness in older children, before closed or open reduction is attempted, as these hips seem more stable after spica cast immobilisation and present with a relatively small incidence of ON [3, 5, 6, 11].

In concordance with the significant difference between Graf type III and IV hips in their chance for successful reduction after Pavlik treatment, we also found that only very few of the Tönnis type 3-4 hips could be reduced successfully by Pavlik harness [10, 13]. This finding was supported by the additional differentiation between subluxed and dislocated hips using the frog-leg or Lauenstein AP pelvic radiograph as all children with a subluxed hip were successfully reduced into stable hips using the Pavlik harness. This is in line with research presented by Bolland et al., as they found a significantly higher number needing closed and open reductions between Tönnis type 4 and Tönnis type 2 groups (OR 4.85, *p* < 0.001) [1].

Pre- and post-treatment AI were also in concurrence with recent literature. As most reports have used ultrasound in the initial screening for DDH it is difficult to compare the initial AI. There was no significant correlation between the initial AI and the success for Pavlik use or risk for ON in our study population. Although the AI was again significantly reduced after the abduction brace was weaned off, when compared to the AI after Pavlik treatment was discontinued, its additional use remains debatable [10, 13].

## Conclusion

The main goal of this study was to evaluate the results of the Pavlik harness as a treatment for late-diagnosed hip dislocation. We conclude that the prolonged use of the Pavlik harness is considered safe, as no inferior dislocation after the prolonged use of the Pavlik was found, and it was successful in infants older than five months with a relatively mild dislocation (<Tönnis type 3). Use of an abduction brace after initial treatment can be considered useful in those patients whose hips are reduced but remain dysplastic when they have been weaned off the Pavlik harness.
